# Novel ZnO microflowers on nanorod arrays: local dissolution-driven growth and enhanced light harvesting in dye-sensitized solar cells

**DOI:** 10.1186/1556-276X-9-183

**Published:** 2014-04-15

**Authors:** Hao Lu, Kaimo Deng, Zhiwei Shi, Qiong Liu, Guobin Zhu, Hongtao Fan, Liang Li

**Affiliations:** 1 School of Physical Science and Technology, Jiangsu Key Laboratory of Thin Films, Collaborative Innovation Center of Suzhou Nano Science and Technology, Soochow University, Suzhou 215006, People’s Republic of China; 2 China National Academy of Nanotechnology and Engineering, Tianjin 300457, People’s Republic of China

**Keywords:** ZnO, Nanorod arrays, Microflowers, Dye-sensitized solar cells, Light harvesting

## Abstract

ZnO nanostructures were manipulated, *via* a low-temperature solution process, from pure nanorod arrays to complex nanostructures of microflowers on nanorod arrays with adjusted quantities of flowers. We proposed the mechanism of local dissolution-driven growth to rationally discuss the novel growth process. These nanostructures were used as photoanodes in dye-sensitized solar cells. Compared to pure nanorod arrays, the nanorod array-microflower hierarchical structures improved the power conversion efficiency from 0.41% to 0.92%, corresponding to a 124% efficiency increase. The enhancement of the efficiency was mainly ascribed to the synergistic effect of the enhanced surface area for higher dye loading and the improved light harvesting from efficient light scattering. Present results provide a promising route to improve the capability of light-harvesting for ZnO nanorod array-based DSSCs.

## Background

Dye-sensitized solar cells (DSSCs) have been regarded as one of the most promising alternatives to silicon solar cells in renewable-energy research based on their special features, such as easy preparation process, low production costs, and relatively high conversion efficiencies [1]. One of the key considerations in fabricating efficient DSSCs is manipulating the structures of photoanodes to enable fast electron transport, effective light harvesting and high dye loading [2-4]. In conventional TiO_2_-disordered nanoparticle-network photoanodes, a high-charge recombination loss limits the conversion efficiency to some degree due to the electron trapping and scattering at grain boundary as well as inefficient light-scattering ability within small-sized nanoparticles. A promising strategy for improving electron transport in DSSCs is to replace the nanoparticle materials of photoanodes by one-dimensional (1D) single-crystalline nanostructures such as nanorods, nanotubes, and nanowires [5-8], which provide a direct conduction pathway for the rapid collection of photogenerated electrons without strong scattering transport.

ZnO, as a wide-bandgap (ca. 3.37 eV) semiconductor, possesses an energy-band structure and physical properties similar to those of TiO_2_ but has higher bulk electronic mobility (205 to 300 cm^2^ · V^−1^ · s^−1^) than TiO_2_ (0.1 to 4.0 cm^2^ · V^−1^ · s^−1^) that would be favorable for electron transport [9-11]. Therefore, ZnO nanorod/nanowire arrays have been extensively studied and are expected to significantly improve the electron diffusion length in the photoanode films [12-17]. Unfortunately, the insufficient surface area of simple 1D nanostructures constrains the energy conversion efficiency to relatively low levels, which was mainly caused by the weak capability of dye loading and light harvesting. One effective strategy to overcome these problems is to utilize ultra-long ZnO nanowires to enhance amounts of dye loading [18,19], and the branched microflowers to strengthen light scattering [20]. However, the ultralong nanowires largely prolonged the distance of electron transport and thus increased the possibility of electron recombination, and the microflowers weakened the advantages of 1D direct transport pathway. Typically, these nanostructures were directly grown on the ZnO seed-coated fluorine-doped tin oxide (FTO) substrates *via* a widely used low-temperature hydrothermal process. Although the synthesis conditions were similar, different morphologies were obtained. The growth process is still not very clear up to now, which emphasizes the need for further systematic investigation of the formation mechanism. In terms of high efficient DSSCs, if we can rationally design a composite structure composed of microflowers and short nanorod arrays, utilizing the synergistic effect of high light harvesting and fast electron transport, the conversion efficiency of DSSCs may be largely improved compared with photoanodes using nanorod arrays or microflowers alone.

In this paper, we demonstrated a novel structure transition from ZnO nanorod arrays to microflowers on nanorod arrays grown on FTO substrates by simply controlling the reaction time. A local dissolution-driven growth mechanism was proposed based on our systematic observation. Considering the respective advantage of nanorod arrays and branched microflowers in the electron transport and light harvesting, we used their synergistic effects in photoanodes to largely improve the efficiency of light harvesting without sacrificing fast electron transport, exhibiting a markedly enhanced power conversion efficiency of 0.92%, which corresponds to an approximately 124% increase as compared to low efficiency of 0.41% for the DSSCs fabricated using simple ZnO nanorod arrays.

## Methods

ZnO nanostructures were grown by a two-step process. First, the ZnO seed layer was formed by spin coating of 5-mM zinc acetate dihydrate (Zn(CH_3_COO)_2_ · 2H_2_O, 98%, Aldrich, St. Louis, MO, USA) ethanol solution onto the FTO substrate, followed by annealing at 400°C for 60 min. ZnO nanostructures were prepared on FTO glass in a 150-ml solution mixture of 25-mM zinc nitrate hexahydrate (Zn(NO_3_)_2_ · 6H_2_O, Aldrich, 98%), 25-mM hexamethylenetetramine (HMTA, Aldrich, 99%) and 2-mM ammonium hydroxide (NH_4_OH, Aldrich, 28%) at 90°C for 30 min to 5 h. FTO substrate with the ZnO seed layer was floated face-down in a closed bottle. Upon completion of the reaction, the substrate was rinsed with deionized water and dried at 60°C overnight and then heated at 420°C for 120 min.

The prepared ZnO nanostructured electrodes were immersed in an ethanol solution containing 0.5 mM of N719 dye (cisbis(isothiocyanato) bis (2,2′-bipyridyl-4,4′-dicarboxylic acid) ruthenium(II)) (Solaronix) at 50°C for 60 min, followed by rinsing in ethanol to remove any dye absorbed physically and drying in air.

Each sensitized electrode was sealed against a counter electrode. The counter electrode was prepared by spreading a droplet of 0.5 mM of chloroplatinic acid (H_2_PtCl_6_ · 6H_2_O, Aldrich, 99.9%) in 2-propanol (Aldrich, 99.7%), which was heated at 350°C for 30 min. The dye-coated electrode and Pt counter electrode were separated with a hot melt plastic frame (Solaronix, Meltonix 1170, 60-μm thick) at pressure of 2.5 bar and temperature of about 105°C. The electrolyte (0.1 M LiI, 0.03 M I_2_, 0.5 M tetrabutylammonium iodide, and 0.5 M 4-*tert*-butylpyridine in acetonitrile) was introduced into the gap formed by two electrodes. The holes were then sealed using hot-melt plastic and a thin glass cover slide. The DSSC active area was 0.15 cm^2^.

The surface and cross-sectional images of ZnO nanostructures were characterized using a field emission scanning electron microscope (FE-SEM, Hitachi S4700, Chiyoda-ku, Japan). The microstructure of ZnO nanorods and microflowers was measured by transmission electron microscopy (TEM) and high-resolution TEM (HRTEM) together with selected-area electron diffraction (SAED). The X-ray diffractometer (XRD) was used to evaluate the phase of products. Photocurrent-voltage (*J*-*V*) was measured by using a Keithley 2400 source/meter controlled by a PC, while irradiating at 100 mW · cm^−2^ (1 sun) with AM 1.5G simulated sunlight produced by a class 3A solar simulator (Newport, 94043A, Irvine, CA, USA). Incident photon-to-electron conversion efficiency (IPCE) was measured as a function of wavelength from 400 to 800 nm under short circuit conditions (Newport, IQE-200). Both the absorption spectrum of the dye and diffuse reflectance spectrum of nanostructures were characterized by a UV-vis spectrophotometer (Shimadzu UV-3600, Kyoto, Japan). The electrochemical impedance spectroscopy (EIS) was measured by an Autolab electrochemical workstation (PGSTAT 302 N) under the open circuit (*V*_oc_) condition in dark. The magnitude of the alternative signal was 10 mV.

## Results and discussion

**Figure 1:**
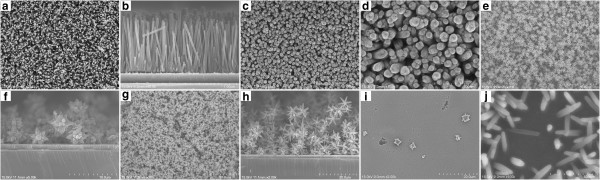
**Top view and cross-sectional SEM images of ZnO nanostructures synthesized at different reaction times. (a,b)** Pure nanorod array at 30 min. **(c,d)** Pure nanorod array with etched hole on top of each nanorod at 40 min. Fewer and multilayers of microflowers on nanorod array at **(e,f)** 1.5 h and **(g,h)** 3 h, respectively. **(i)** Nanorod array with microflowers etched away and **(j)** nanorods with shortened length at 5 h.

Figure 1 shows the representative SEM images of ZnO nanostructures synthesized at different reaction times from 30 min to 5 h. When the reaction time was 30 min, the vertically oriented nanorod array with an average length of 1.5 μm and a diameter of 80 nm was obtained (Figure 1a,b). After 40 min of reaction, the basic morphology of array was preserved, but the close examination revealed that a central hole lay on every top plane of the nanorods (Figure 1c,d). This implies that a dissolution process may occur during the growth. As the reaction time was prolonged to 1.5 h, the sample was composed of microflowers on the top and a nanorod array underneath (Figure 1e,f). With increasing the reaction time to 3 h, multilayers of microflowers were formed, which makes the nanorod array invisible (Figure 1g,h). Further extending the reaction time to 5 h, unexpectedly, the microflowers almost completely disappeared and large etched pits on the surface appeared, and even the length of nanorods was reduced significantly to about 300 nm (Figure 1i,j).

**Figure 2:**
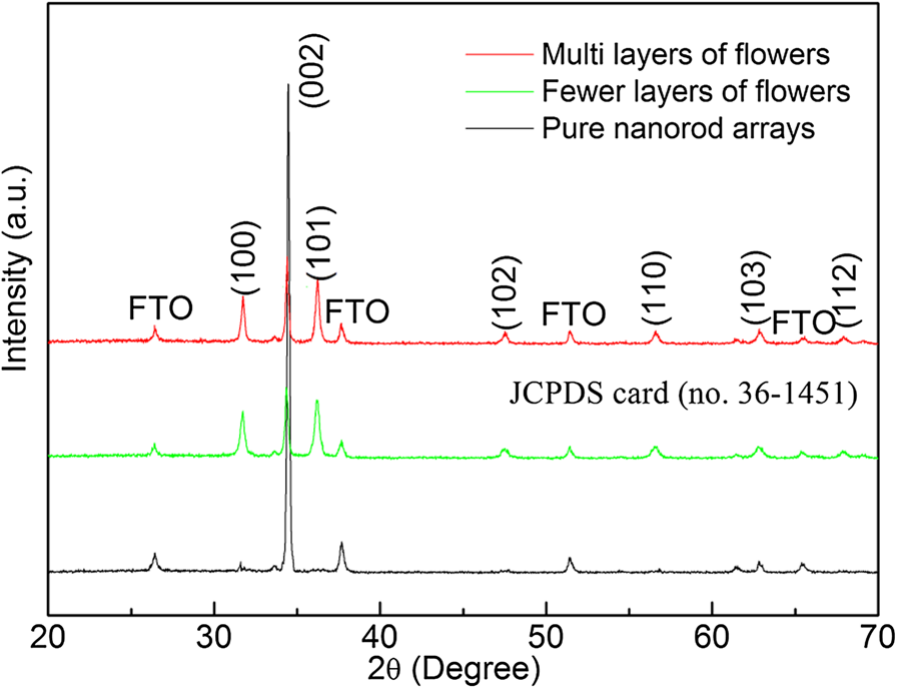
XRD pattern of as-prepared ZnO pure nanorod arrays and fewer and multilayers of microflowers on nanorod arrays.

**Figure 3:**
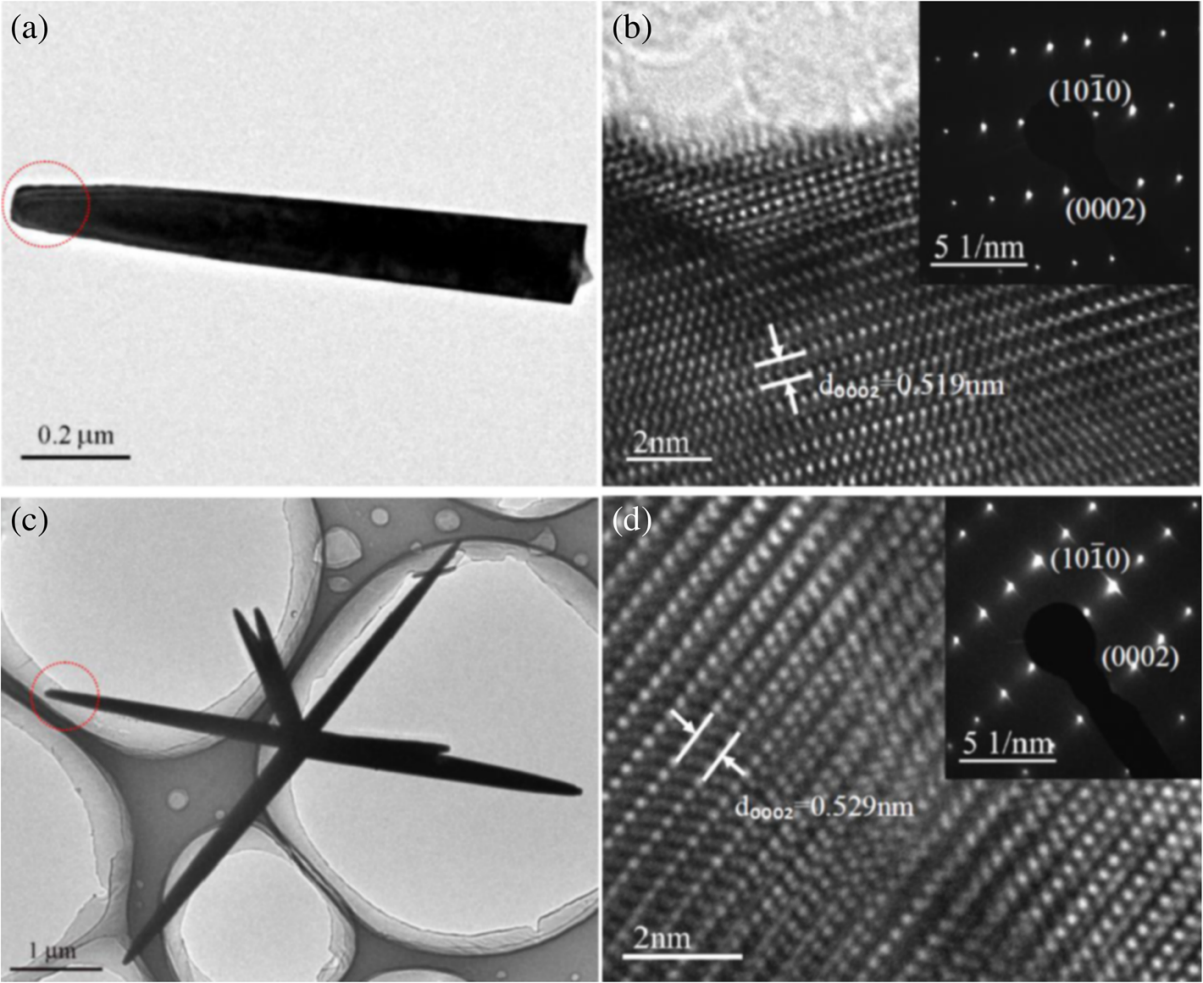
TEM (a,c) and HRTEM images (b,d) of ZnO nanorods and microflowers, respectively.

The phase of as-prepared nanostructures was characterized by XRD pattern, as shown in Figure 2. All diffraction peaks can be indexed to the hexagonal wurtzite phase of ZnO (JCPDS Card No. 36–1451) with not any impurities. The strong relative intensity of the (0002) diffraction peak reveals a texture effect of the arrays consistent with *c*-axis-oriented nanorods, which will be further confirmed by TEM images (Figure 3). Figure 3a shows a typical TEM image of ZnO nanorod scratched from the ZnO nanorod array on a FTO substrate. Corresponding HRTEM image and SAED pattern (Figure 3b), taken from the red circled area in Figure 3a, exhibit that ZnO nanorod is a single crystal with the preferential [0001] growth direction. Figure 3d illustrates the HRTEM image and SAED pattern of ZnO nanorod, a random branch of microflower as shown in Figure 3c, revealing that the growth direction of single crystal is also along [0001].

Based on the above growth phenomena, we propose a local dissolution-driven growth mechanism for present ZnO nanostructures. As we know, an alkaline solution is essential for the formation of ZnO nanostructures because normally divalent metal ions do not hydrolyze in acidic environments. In our experiments, both HMTA and NH_3_ · H_2_O provided the NH_3_ (NH^4+^) and OH^−^, and the NH_3_ served as the complex agent to form zinc amino complex [Zn(NH_3_)_4_]^2+^ with Zn^2+^, according to [21-24].

(1) $$\mathrm{HMTA}+6H_{2}O↔4NH_{3}+6\mathrm{HCHO}$$

(2) $$NH_{3}+H_{2}O↔N{H_{4}}^{+}+OH^{-}$$

(3) $$Zn^{2+}+4NH_{3}↔{\left[\mathrm{Zn}{\left(NH_{3}\right)}_{4}\right]}^{2+}$$

In the initial reaction stage, the Zn^2+^ supplied from the decomposition of [Zn(NH_3_)_4_]^2+^ reacted with OH^−^ and Zn(OH)_2_ colloids formed in the solution (reaction 4), and part of Zn(OH)_2_ colloids dissolved into Zn^2+^ and OH^−^ because the precipitates of Zn(OH)_2_ are more soluble as compared to the ZnO precipitates (reaction 5). When the concentration of Zn^2+^ and OH^−^ reached the supersaturation degree of ZnO, ZnO nuclei formed (reaction 6) and acted as building blocks for the formation of final products. The growth units of [Zn(OH)_4_]^2−^ formed according to reaction 7 [25-27].

(4) $$Zn^{2+}+2OH^{-}↔\mathrm{Zn}{\left(\mathrm{OH}\right)}_{2}$$

(5) $$\mathrm{Zn}{\left(\mathrm{OH}\right)}_{2}\to Zn^{2+}+2OH^{-}$$

(6) $$Zn^{2+}+OH^{-}↔\mathrm{ZnO}+H_{2}O$$

(7) $$\mathrm{Zn}{\left(\mathrm{OH}\right)}_{2}+2OH^{-}↔{{\left[\mathrm{Zn}{\left(\mathrm{OH}\right)}_{4}\right]}^{2}}^{-}$$

Wurtzite structured ZnO, which is confirmed by the XRD pattern (Figure 2), grown along the *c*-axis has high-energy polar surfaces such as ± (0001) surfaces with alternating Zn^2+^ terminated and O^2−^ terminated surfaces [28]. Therefore, when a ZnO nucleus was newly formed, the incoming precursor molecules tended to favorably adsorb on the polar surfaces, leading to a fast growth along the [0001] direction (Figure 3a,b) and thus 1D nanorod structure formed. In addition, attachment of HMTA to the nonpolar side facets also facilitated the anisotropic growth in the [0001] direction [29]. With increasing the reaction time to 40 min, two phenomena may occur simultaneously in the high pH solution (pH = 10.0): (1) The Zn^2+^ was consumed quickly, prohibiting or slowing down the growth of ZnO nanorods; (2) Laudise et al. reported that the higher the growth rate, the faster the disappearance of a plane [30]. Here, the (0001) plane, the most rapid growth rate plane, dissolved more quickly than the other six symmetric nonpolar planes in the growth process, which is confirmed by the formed holes on the top plane of nanorods. The preferential formation of holes on top surface of ZnO is related to its crystallographic characteristics of surface polarity and chemical activities, which is caused by the more reactive {0001} faces with a higher surface energy/atomic density than for the other faces. On the other hand, the dissolved Zn^2+^ from nanorods caused local supersaturation around the top surface of nanorods and favored new nucleation. The shape of the final crystal was mainly determined by the distribution of active sites on the surfaces of the nuclei. In the high pH environment, large quantities of growth units of [Zn(OH)_4_]^2−^ were adsorbed on the circumference of the ZnO nuclei and the surface energy of ZnO nuclei decreased, resulting in the multiple active sites generated on the surface. Subsequently, ZnO crystals can present spontaneously preferential growth along the [0001] direction (Figure 3c,d) from these active sites due to the anisotropic growth habit of ZnO, and gave the nanorod-based flower-like form. Once the Zn^2+^ was consumed severely, the growth speed reduced greatly and the etching process dominated. As the reaction time was long enough, up to 5 h, all the microflowers almost disappeared and nanorods also became shorter due to etching.

**Figure 4:**
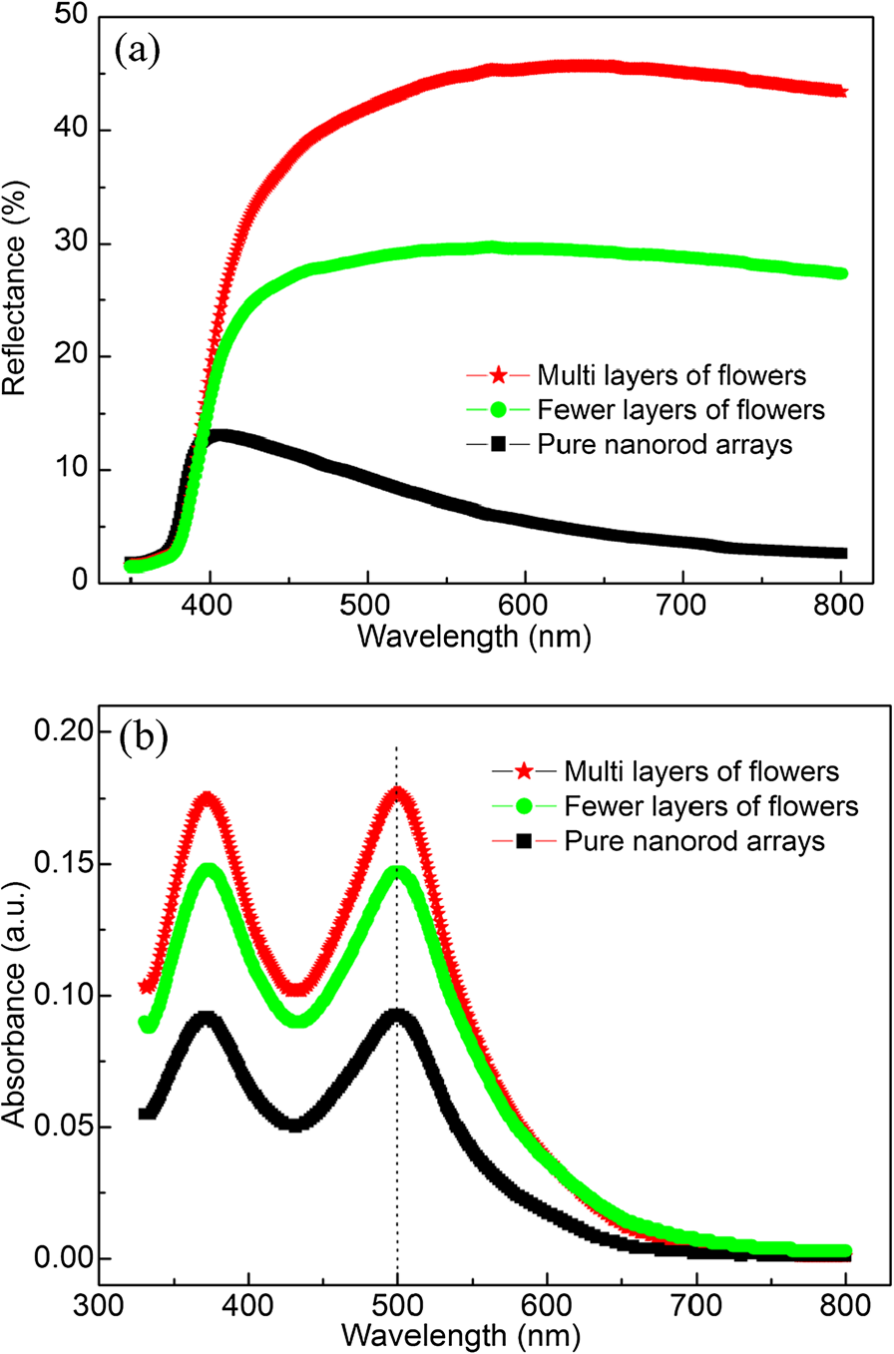
**Diffusion reflectance spectra (a) and dye absorption spectra (b) of photoanodes.** With pure nanorod arrays and fewer and multilayers of microflowers on nanorod arrays.

The key to highly efficient DSSCs lies in a large amount of dye adsorption, sufficient light harvesting and fast charge transport. The UV-visible diffuse reflectance spectra of ZnO photoanodes were measured, as shown in Figure 4a. The pure nanorod arrays showed little diffuse reflectance (10% at 400 nm), and a rapid decrease in diffuse reflection capacities were observed as the visible wavelength increased from 400 to 800 nm. A higher reflectance value close to 30% was obtained for composite nanostructures of nanorods and fewer layers of microflowers (fewer layers means that microflowers just cover the whole surface of nanorod arrays) in the range of 400 to 800 nm. The reflectance ability of composites continuously increased with the layer of microflowers and the maximum value can be as high as 46%, which provides a basis for the effective use of long wavelength photonic energy. Thus, composite nanostructures could extend the photoresponse of the photoanode well into the visible spectrum, resulting in an enhancement of light utilization efficiency. The capacity of dye loading had a profound influence on the photocurrent density. The amount of adsorbed N719 dye was estimated by measuring the eluted dye molecules from samples with UV-vis absorption spectroscopy (Figure 4b). To measure the amount of adsorbed dye in a photoanode, 0.5-mM dye was dissolved in 10-mM NaOH for reference. Dye-absorbed photoanodes were placed in 4 mL of 10-mM NaOH in water until the dye was completely desorbed from the electrode. The absorption value at 500 nm was used to calculate the number of absorbed dye molecules according to the Beer-Lambert law, *A* = *ϵlc*, where *A* is the absorbance at 510 nm, *ϵ* = 8,176/Mcm is the molar extinction coefficient of the dye at 500 nm, *l* is the path length of the light beam (1.0 cm), and *c* is the dye concentration. The amounts were 23.4, 26.9, and 44.3 nmol · cm^−2^ for pure nanorod array and composite nanostructures with fewer and multilayers of microflowers (multilayers means higher quantity of microflowers compared with that of fewer layers), respectively. Clearly, the composite nanostructures with fewer and multilayers of microflowers showed 1.1 and 1.9 times higher dye loading than pure nanorod arrays.

**Figure 5:**
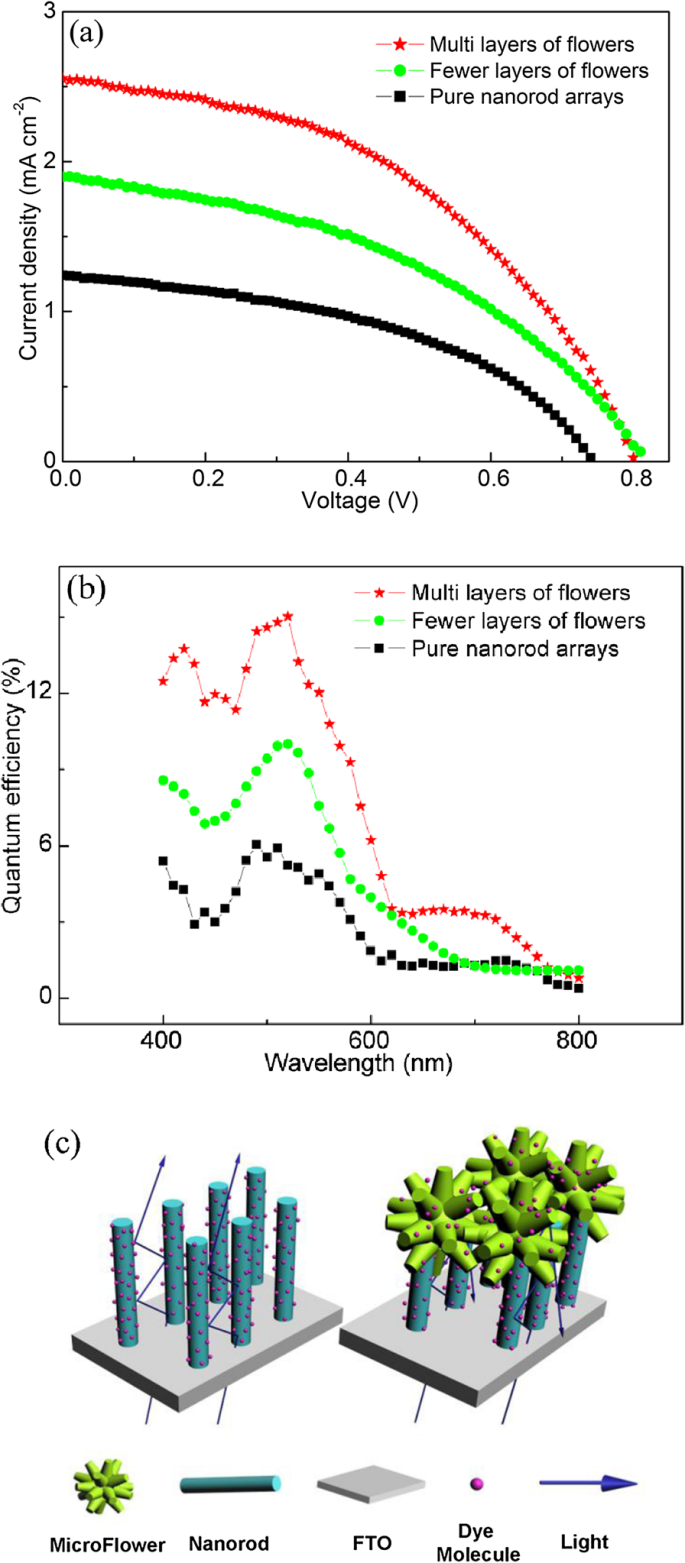
**Photocurrent-photovoltage ( *J-V *) curves (a) and IPCE spectra (b) for DSSCs and schematic of characteristics of light (c). (a,b)** With pure nanorod arrays, fewer and multilayers of microflowers on nanorod arrays as photoaonodes.

Figure 5a presents the current density-voltage (*J*-*V*) curves of DSSCs fabricated with the ZnO nanostructures as photoanodes. Cell performance including open-circuit voltage (*V*_oc_), short-circuit current density (*J*_sc_), fill factor (*FF*), and an energy conversion efficiency (*η*) are summarized in Table 1. It shows that DSSC with the pure nanorod array (average thickness of 1.5 μm) as a photoanode possesses an efficiency of 0.41%, which is comparable to those with a larger thickness of 7 (0.45%) and 8 μm (0.3%) in reported results [31,32]. The conversion efficiency of cell with fewer and multilayers of microflowers as photoanode is 0.65% and 0.92%, respectively, which is approximately a 58% and 124% enhancement over that of the pure nanorod array cell. The IPCE is determined by the light absorption efficiency of the dye, the quantum yield of electron injection and the efficiency of collecting injected electrons at the FTO substrate, which are strongly affected by the photoanode properties of DSSCs. Compared with the pure nanorod array and composite structure with fewer layers of microflowers, the composite structure with multilayers of microflowers has a higher IPCE over the whole range from 400 to 800 nm (Figure 5b). At the maximum value of the IPCE spectra at about 500 nm, the IPCE of the multilayers of microflowers was approximately 15.0%, obviously higher than those of the pure nanorod array (6.0%) and fewer layers of microflowers (10.0%).

**Table 1:** Device performance of DSSCs with photoanodes of different geometries

**Sample**	***J***_**sc **_**(mA · cm**^**−2**^**)**	***V***_**oc **_**(V)**	***FF***	***η***	**Absorbed dye (nmol · cm**^**−2**^**)**
Pure nanorod arrays	1.24	0.78	45.52	0.41	23.4
Fewer layers of microflowers on nanorod arrays	1.94	0.82	42.33	0.65	26.9
Multilayers of microflowers on nanorod arrays	2.62	0.84	45.33	0.92	44.3

Data were taken from *J*-*V*, IPCE, and dye absorption curves.

Improved cell performance mostly results from the enhancement of the *J*_sc_ value, as the *V*_oc_ and *FF* values are not significantly changed (Table 1). The increased *J*_sc_ is contributed by a well developed light scattering structure related with efficient light harvesting and larger surface area related with higher dye loading, as schematically shown in Figure 5c. For the pure nanorod arrays, the unabsorbed light will penetrate through the photoanode without being scattered back to enhance light absorption, and the amount of dye loading is low due to their small surface area. Concerning the advantages of microflowers on nanorod arrays, the microsized branched microflowers not only multireflect but also scatter the incident light of different wavelengths in the whole range of visible light. In addition, this composite nanostructure will provide additional surface area to absorb more dye. Therefore, the bi-functional photoanode materials are featured with increased dye loading rate and maximized absorption of light in the range of 400 to 800 nm, greatly enhancing the light harvesting efficiency.

**Figure 6:**
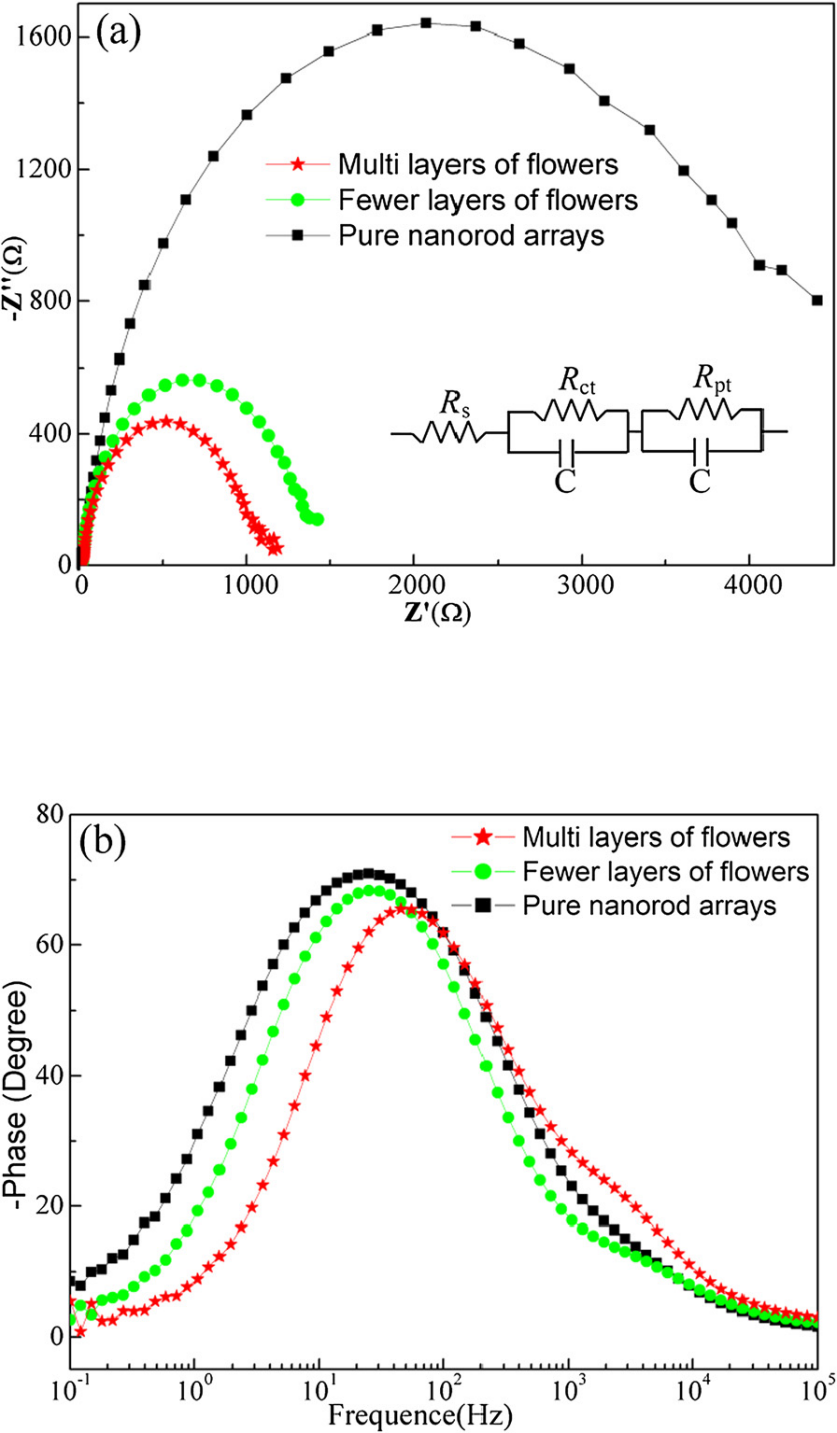
**EIS results: (a) Nyquist plots and (b) Bode phase spectra.** The inset in **(a)** shows the equivalent circuit model.

Electrochemical impedance spectroscopy (EIS) was measured to identify the charge-related transport and recombination in electrodes and interfaces. Figure 6a shows the Nyquist plots which were fitted by the classical model of equivalent electrical circuit (the inset at the bottom-right corner). The size of semicircle in the intermediate frequency range (ca. 1 to 1,000 Hz) represents the electron transfer resistance at the ZnO/dye/electrolyte interface (*R*_ct_), indicating that the recombination becomes serious gradually from pure nanorod arrays to fewer and multilayers of microflowers. From the Bode spectrum (Figure 6b), the lifetime of injected electrons (*τ*_n_) was calculated from the peak frequency (*f*_max_) in the middle frequency range based on the relationship *τ*_n_ = 1/ 2π*f*_max_. The electron lifetime in three types of electrodes is 6.1, 5.8, and 3.0 ms for pure nanorod arrays and fewer and multilayers of microflowers, respectively, which suggests that electrons can transport effectively in three nanostructures without large difference, although their recombination is different.

## Conclusions

We present a highly efficient and pure light harvesting strategy by fabricating novel composite nanostructured photoanodes to improve the energy conversion efficiency of DSSCs. The ZnO nanostructures were manipulated from pure nanorod arrays to composite nanostructures of microflowers on nanorod arrays with adjusted quantities of flowers. The plausible mechanism of local dissolution-driven growth was proposed. Such composite nanostructures were then exploited as photoanodes of DSSCs to yield largely enhanced efficiency of 0.92%, as compared to a low efficiency of 0.41% for the DSSCs prepared by using a pure ZnO nanorod array, corresponding to a 124% efficiency increase. The improved performance is a direct consequence of the synergistic effect of the enhanced surface area for higher dye loading, the improved light harvesting from efficient light scattering, as well as the fast carrier transport facilitated by continuous growth between microflowers and nanorods. From present results, the conversion efficiency of ZnO-based DSSCs can be further improved by constructing more complex nanostructures in the future.

## Competing interests

The authors declare that they have no any competing interests.

## Authors’ contributions

HL participated in the design of experiments and drafted the manuscript. KD participated in the analysis of TEM and IV data. ZS participated in the experiment of XRD and data analysis. QL participated in the analysis of IV and SEM. GZ participated in the collection of SEM and analysis of data. HF participated in the collection of HRTEM and analysis of data. LL participated in the design and analysis of data and revision of manuscript. All authors read and approved the final manuscript.
